# Single nucleotide polymorphism analysis of *pvmdr-1* in *Plasmodium vivax* isolated from military personnel of Republic of Korea in 2016 and 2017

**DOI:** 10.1186/s12936-022-04214-6

**Published:** 2022-06-28

**Authors:** Jin-Jong Bong, Wonsig Lee, Chan Hee Lee, Quehn Park, Kyung Tae Noh

**Affiliations:** Department of Infectious Disease Research, Armed Forces Medical Research Institute, 90bun, Jaunro, Yuseong-gu, Daejeon, 34059 Republic of Korea

**Keywords:** *Plasmodium vivax*, Multi-drug resistance protein 1, Single nucleotide polymorphism, Chemoprophylaxis

## Abstract

**Background:**

Malaria chemoprophylaxis using chloroquine (CQ) and primaquine (PQ) has been administered to resident soldiers in the 3rd Army of Republic of Korea (ROK) to prevent malaria infection since the year 1997. Due to mass chemoprophylaxis against malaria, concern exists about the occurrence of chloroquine resistance (CQR). This study aimed to investigate the single nucleotide polymorphisms (SNPs) of the *Plasmodium vivax* multi-drug resistance protein-1 (*pvmdr-1*) gene to monitor the risk of CQR.

**Methods:**

SNPs of the *pvmdr-1* gene were analysed in 73 soldiers of the 3^rd^ Army of ROK diagnosed with infection by *P. vivax*.

**Results:**

Quintuple mutations (G698S, L845F, M908L, T958M, and F1076L) were detected in 73 soldiers. A newly identified non-synonymous mutation in the Y541C position had been introduced into *P. vivax* malaria-endemic areas in ROK, at a frequency of 1.3% (1/73). In addition, synonymous mutations were detected at positions K44 (38.4%, 28/73), L493 (26%, 19/73), T529 (61.6%, 45/73), and E1233 (52.1%, 38/73). Based on these SNPs, *pvmdr-1* sequences of ROK were classified into 6 haplotypes. The phylogenetic analysis closed to the type of North Korean showed that *P. vivax* malaria of ROK could be a reason of influx from North Korea.

**Conclusions:**

This study showed that synonymous and non-synonymous mutations of *pvmdr-1* were observed in the malaria chemoprophylaxis-executed regions of ROK from 2016 to 2017. Based on the rapid transition of *pvmdr-1* SNPs, continuous surveillance for SNPs of *pvmdr-1* related to CQR in the malaria-endemic regions of ROK is essential.

**Supplementary Information:**

The online version contains supplementary material available at 10.1186/s12936-022-04214-6.

## Background

Malaria, a life-threatening disease caused by *Plasmodium* parasites*,* endangers about 40% of the world's population [1, 2]. Depending on the recent World Health Organization (WHO) malaria report, all malaria infection cases have been declined from 238 million cases in 2000 to 229 million cases in 2019. In case of *P. vivax* malaria, it decreased from about 7% in 2000 to 3% in 2019 [3]. This worldwide malaria reduction trends are attributed to the WHO preventive policy of eradicating malaria. Nevertheless, annually, more than 300 million people of the world`s population are infected with malaria, and about 500,000 people die from malaria. Vivax malaria was supposed to be eradicated in the Republic of Korea (ROK), but a re-emergence was reported in Paju City of Gyeonggi Province in 1993 [4, 5]. According to Korea Disease Control and Prevention Agency, since re-emergence occurred in a soldier of the 3rd Army of ROK, the incidence of malaria infections has been steadily increasing [6]. In ROK, *P. vivax* malaria-endemic regions are localized near the DMZ (demilitarized zone; the border between ROK and the Democratic People’s Republic of Korea, DPRK) [7]. Thus, soldiers and civilians residing in DMZ have been classified as a high-risk group of malaria infection. Among the total malaria patients of ROK, the military (soldiers and military veterans) accounted for a large proportion. Because of this, members of the army of ROK have undergone prophylactic chemotherapy against malaria to prevent patient outbreaks since 1997 [8]. The detailed procedure of chemoprophylaxis is as following, 300 mg chloroquine (CQ) is administrated weekly to military personnel nearby DMZ from July to October for 15 weeks, and 15 mg PQ is subsequently administrated daily for 2 weeks [9].

CQ is effective to eradicate *P. vivax* in asexual blood stages and gametocytes, and primaquine (PQ) is responsible for killing the hypnozoite form of *P. vivax* in the liver stage. Due to the risk of chloroquine resistance (CQR) by massive and long-term use of chemoprophylaxis for prevention of malaria [10], the army of ROK has been monitoring drug resistance by analysing mutations in *pvmdr-1* [11]. Subsequently, several changes in the ROK Armed Forces chemoprophylaxis programme were implemented, including the reduction of the period of hydroxychloroquine (400 mg weekly) chemoprophylaxis by 2 months in 2008, and the discontinuation of terminal primaquine chemoprophylaxis (15 mg × 14 days) in 2016 in moderate-risk area [12]. To date, various CQR cases in several regions including Indonesia, Southeast Asia, India, and Central and South America have been reported [13–19]. There is concern about the influx of malaria resistance from Southeast Asian countries [20]. Recent studies have been shown that long-term chemoprophylaxis could induce genetic mutation. For example, polymorphism of *pvcrt-o* and *pvmdr-1* was detected by CQ treatment [21–23]. Thus, it can be hypothesized that genetic changes occur in malaria chemoprophylaxis-executed malaria-endemic regions of ROK. Herein, this study was to focus on the surveillance of genetic mutation in *pvmdr-1* (Malaria drug resistance-related gene).

## Methods

### Ethics statement and sample preparation

This study was approved by the ethics committee of the Armed Forces Medical Command (Approval No. AFMC-16067-IRB-16-056, September 2016). An approval form was used to obtain written informed consent and permission from each participant for providing a 5 ml blood sample.

### Volunteers enrolment—inclusion criteria

Blood samples were collected from malaria patients who agreed to the study. Positive patients with a rapid diagnostic test (Standard Diagnostics Inc., USA) or blood smear test with microscopy, or malaria-suspected patients (A person who needs a malaria molecular test as a result of medical treatment with a body temperature of 38 degrees Celsius) were enrolled this study. Most patients have a history of fever in the previous 48 h without malaria chemoprophylaxis compliance. All blood samples collected from 3 military hospitals (Yangju, Koyang, and Ildong), during 2016–2017 were screened using *18S rRNA* nested PCR.

### Collection of clinical isolates

141 venous blood samples (Whole blood-EDTA samples) from male patients infected with malaria or malaria suspected patients with fever were collected in the three Armed Forces Hospitals (Yangju, Koyang, and Ildong) near the DMZ located in northern Gyeonggi Province and northwest region of the ROK from 2016 to 2017. The Armed Forces Hospitals performed a rapid diagnostic test (STANDARD DIAGNOSTICS Inc., USA), and a blood smear test with microscopy for all participants.

### *Plasmodium vivax* nested PCR analysis (*18S rRNA* and *pvmdr-1*)

Genomic DNA was extracted from 200 ul Whole blood-EDTA using DNeasy Blood and Tissue Kit (Qiagen, Hilden, Germany) as recommended by the manufacturer. For screening *P. vivax* infection, purified DNA samples from all patients were diagnosed by nested polymerase chain reaction (PCR) targeting *P. vivax 18S rRNA*. 1100 bp or 120 bp was detected by 2nd nested PCR [24].

**Fig. 1:**
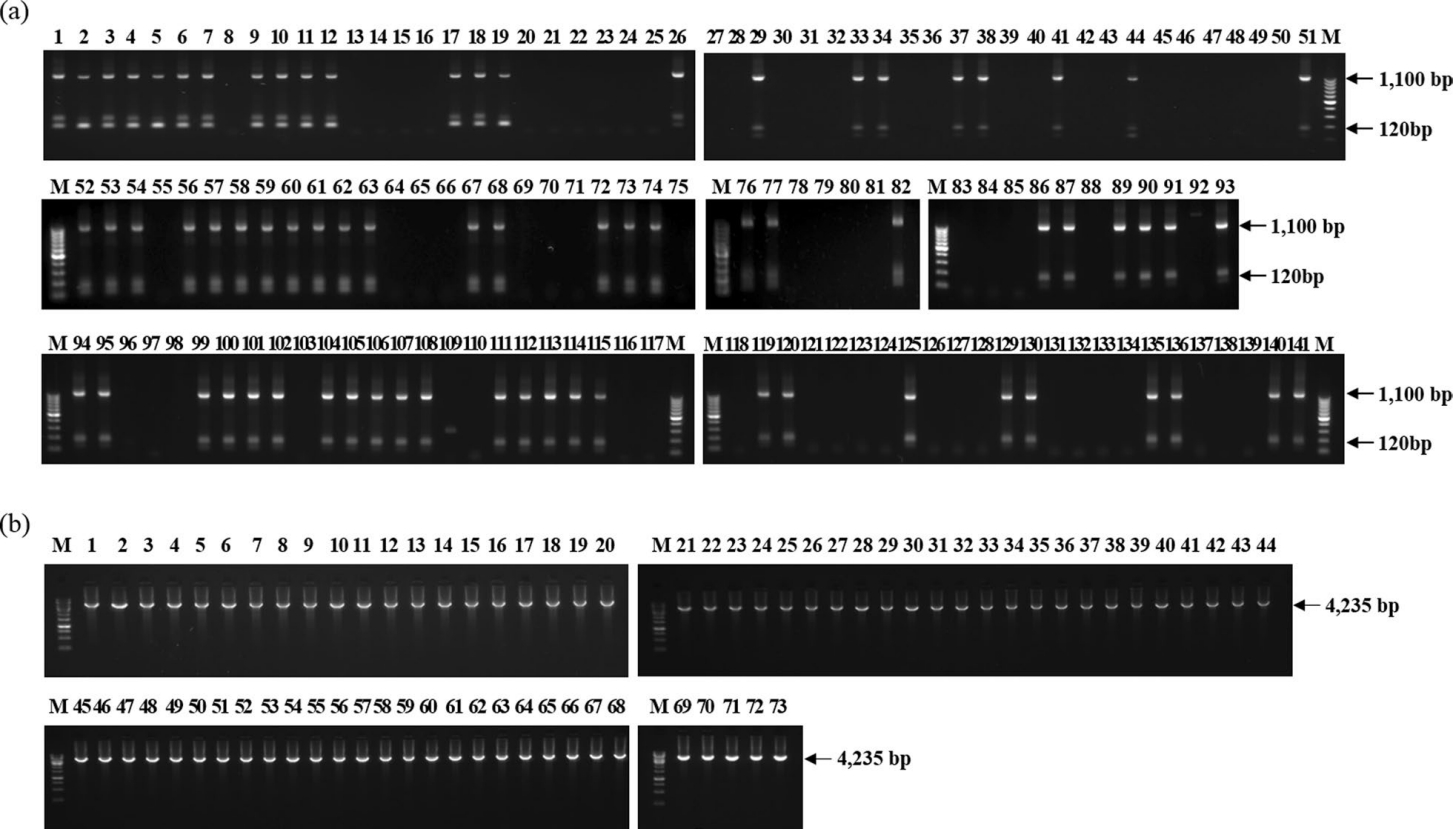
Gel electrophoresis of *P. vivax 18S rRNA* and *pvmdr-1* nested PCR. **a** *18S rRNA* nested PCR. Blood samples were collected from malaria patients who agreed to the study. All blood samples collected in three hospitals (Yangju, Koyang and Ildong), during 2016 and 2017, were screened using *18S rRNA* nested PCR. Based on the screened result, 73 samples were identified as positive samples. 1100 bp or 120 bp was detected by 2nd PCR of *18S rRNA*. **b** Nested PCR targeting *pvmdr-1* amplicons ranged from 104 bp to 4,254 bp were observed in 73 *P. vivax* positive samples using *pvmdr-1* nested PCR. M: 1 Kb Plus DNA Ladder

As shown in the previous report [23], the *pvmdr-1* gene was amplified using the indicated primer sets of nested PCR (Table 1) for the SNPs analysis of *pvmdr-1*. The first round of PCR was performed under the following conditions: 94 °C for 5 min, followed by 30 cycles of 94 °C for 30 s, 57 °C for 30 s, and 72 °C for 4.5 min, and a final extension at 72 °C for 10 min. The second PCR was performed under the following conditions: 94 °C for 5 min, followed by 35 cycles of 94 °C for 30 s, 57 °C for 30 s, and 72 °C for 4 min, and a final extension at 72 °C for 10 min. PCR products were analysed on 1.5% agarose gels using 1 Kb Plus DNA Ladder (Thermo Fisher Scientific) by electrophoresis (Fig. 1) and visualized by Fluor Chem FC3 (Protein Simple, USA).

**Table 1:** Information of primers used to amplify *pvmdr-1* gene

Primers	Sequence (5' → 3')
*Pvmdr-1* (1st F)	ATG AAA AAG GAT CAA AGG CAA C
*Pvmdr-1* (1st R)	CTA CTT AGC CAG CTT GAC GTA C
*Pvmdr-1* (2nd F)	TTG AAC AAG AAG GGG ACG TT
*Pvmdr-1* (2nd R)	CTT ATA TAC GCC GTC CTG CAC
Sequencing primer 1	TAC GGA ACG AGA ATC ATC AT
Sequencing primer 2	GAT GGT CAG CAT GAA CAT AC
Sequencing primer 3	TCA GGT GGA CAG AAG CAG AG
Sequencing primer 4	GCG AAC TCG AAT AAG TAC TC
Sequencing primer 5	AAG TCC CTC ATC GAC GTG AG

*Pvmdr-1* primers set were adapted from Barnadas et al. [23]. Sequencing primers were newly designed for this study

### Sequencing of *pvmdr-1*

After the amplification, sequence analysis was performed by specific sequencing primers as shown in Table 1. Sequencing primers were designed to cover the near full-length *pvmdr-1* gene (4,395 bp). Direct sequencing of PCR products was performed by using Big Dye™ Terminator v3.1 Cycle Sequencing Kit (Applied Biosystems, CA, USA) and the products were resolved on ABI 3730XL Genetic Analyzer (Applied Biosystems, CA, USA).

### Phylogenetic analysis

Nearly full-length sequence (from 104 bp to 4,290 bp) of *pvmdr-1* was used to perform phylogenetic relationship analysis. The sequences of Sal I strain (GenBank Accession# AY571984) and global parasites in PlasmoDB [25] were used as reference sequences. *pvmdr-1* sequences of 73 *P. vivax* clinical samples isolated from military personnel of the ROK army in 2016 and 2017 were analysed using BioEdit Sequence Alignment Editor. The phylogenetic analysis was constructed by 1000 bootstrap replications [26] using the neighbor-joining method [27].

## Results

### Nested PCR and SNP analyses of the *pvmdr-1* gene

A total of 73 out of 141 samples were identified as *P. vivax* infected specimen through nested PCR analysis targeting *18S rRNA* (Fig. 1a). For SNP analysis of *pvmdr-1*, nested PCR targeting *pvmdr-1* was performed using 73 positive specimens (Fig. 1b), which were used for sequence analysis. As shown in Table 2, five non-synonymous mutations (G698S, L845F, M908L, T958M, and F1076L) were detected in all specimens (2016: 20/20, 2017: 53/53). Interestingly, a novel non-synonymous mutation (Y541C) was detected in 1 soldier at a frequency of 1.8% (2017: 1/53). In addition, silent mutations in K44 [2016: 4/20 (20%), 2017: 24/53 (45.2%)], L493 [2016: 5/20 (25%), 2017: 14/53 (26.4%)], T529 [2016: 15/20 (75%), 2017: 30/53 (56.6%)], and E1233 [2016: 8/20 (40%), 2017: 30/53 (56.6%) positions were detected in specimens. Alignment and mapping data of *pvmdr-1* wild-type and mutant-type sequences were provided (Additional File 1: Fig. S1).

**Table 2:** The list and frequency of *pvmdr-1* mutations in ROK army from 2016 to 2017

Mutations in the *pvmdr-1* gene	No.(%) of mutated isolates	
	2016 (N = 20)	2017 (N = 53)
K44 (AA**G**-132-AA**A**)	4/20 (20%)	24/53 (45.2%)
L493 (**T**TA-1477-**C**TA)	5/20 (25%)	14/53 (26.4%)
T529 (AC**A**-1587-AC**G**)	15/20 (75%)	30/53 (56.6%)
**Y541C (TAC-1622-TGC)**	0/20 (0%)	1/53 (1.8%)
G698S (**G**GC-2092-**A**GC)	20/20 (100%)	53/53 (100%)
L845F (**C**TC-2533-**T**TC)	20/20 (100%)	53/53 (100%)
M908L (**A**TG-2722-**C**TG)	20/20 (100%)	53/53 (100%)
T958M (A**C**G-2873-A**T**G)	20/20 (100%)	53/53 (100%)
F1076L (**T**TT-3226-**C**TT)	20/20 (100%)	53/53 (100%)
E1233 (GA**G**-3699-GA**A**)	8/20 (40%)	30/53 (56.6%)

Quintuple non-synonymous mutations (G689S, L845F, M908L, T958M, and F1076L) and synonymous mutations (K44, L493, T529, and E1233) were detected in specimens. Furthermore, a novel non-synonymous mutation (Y541C) was detected in 1 patient

### Genotypic classification of the *pvmdr-1* gene

**Fig. 2:**
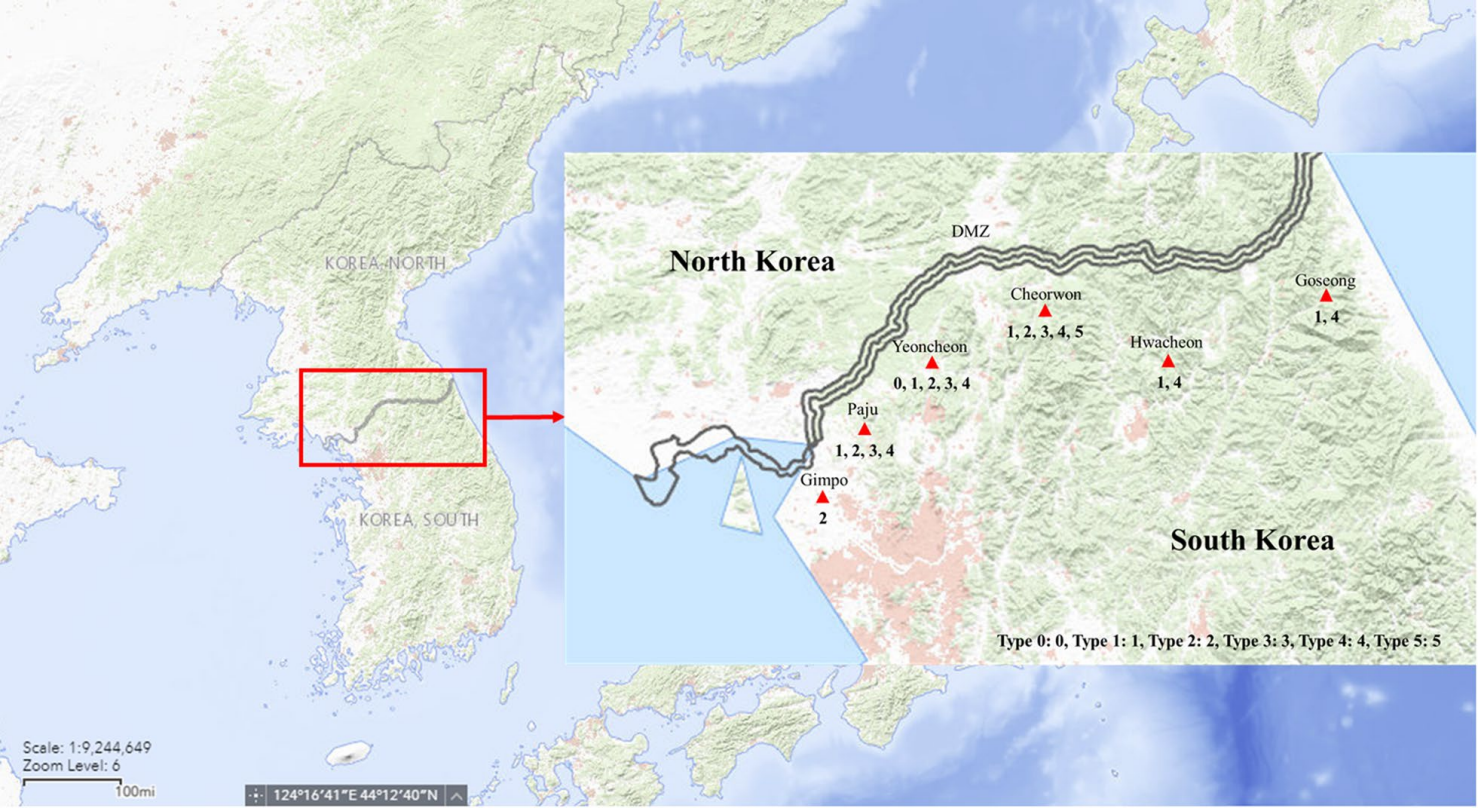
The malaria-endemic region and *pvmdr-1* haplotypes of ROK in 2016 and 2017. Each red triangle indicates malaria-endemic region of haplotypes [Goseong (Type 1 and 4), Cheorwon (Type 1, 2, 3, 4, and 5), Yeonchen (Type 0, 1, 2, 3 and 4), Paju (Type 1, 2, 3 and 4), Gimpo (Type 2), and Hwacheon (Type 1 and 4)]. Six haplotypes were classified from 73 specimens collected in the Armed Forces Hospitals (Yangju, Koyang, and Ildong). Numeric letters mean 6 haplotypes. Reprinted from USGS National Map Viewer [36] under a CC BY license, with permission from U.S. Geological Survey, original copyright 2002

As shown in Table 3, all specimens were clustered into 6 groups from Type 0 to Type 5, and all haplotypes possessed 5 non-synonymous mutations (G698S, L845F, M908L, T958M, and F1076L). 6 haplotypes were classified genotypically based on *pvmdr-1* sequences from 73 specimens in the ROK army from 2016 to 2017 (Fig. 2). Type 0 was found in 1 case in 2016, and there was no mutation other than the 5 non-synonymous mutations. For synonymous mutation, K44 mutant was found in Type 1 (2016; 1/20, 2017; 8/53) and Type 2 (2016; 3/20, 2017; 16/53), T529 mutant was found in Type 3 (2016; 5/20, 2017; 14/53), Type 4 (2016; 10/20, 2017; 14/53), and Type 5 (2016; 0/20, 2017 1/53), E1233 mutant was found in Type 2 (2016; 3/20, 2017; 16/53) and Type 3 (2016; 5/20, 2017; 14/53), and L493 was only found in Type 3 (2016; 5/20, 2017; 14/53). Type 5 (2017; 1/53) was nearly the same as Type 4, except it harboured the first identified Y541C mutation. In the years 2016 and 2017, Type 4 (24/73) was abundant and showed a 100% match with the *pvmdr-1* sequence of North Korean.

**Table 3:** The haplotypes of the *pvmdr-1* gene in Republic of Korea from 2016 to 2017

	K44 AAG → AAA	L493 TTA → CTA	T529 ACA → ACG	Y541C TAC → TGC	**G698S** **GGC** ** → AGC**	**L845F** **CTC** ** → TTC**	**M908L** **ATG** ** → CTG**	**T958M** **ACG** ** → ATG**	**F1076L** **TTT** ** → CTT**	E1233 GAG → GAA	**Frequency**	
											**2016**	**2017**
Type 0	–	–	–	–	**AGC**	**TTC**	**CTG**	**ATG**	**CTT**	–	1	0
Type 1	AAA	–	–	–	**AGC**	**TTC**	**CTG**	**ATG**	**CTT**	–	1	8
Type 2	AAA	–	–	–	**AGC**	**TTC**	**CTG**	**ATG**	**CTT**	GAA	3	16
Type 3	–	CTA	ACG	–	**AGC**	**TTC**	**CTG**	**ATG**	**CTT**	GAA	5	14
Type 4	–	–	ACG	–	**AGC**	**TTC**	**CTG**	**ATG**	**CTT**	–	10	14
Type 5	–	–	ACG	TGC	**AGC**	**TTC**	**CTG**	**ATG**	**CTT**	–	0	1
											20	53

All haplotypes (Type 0 to Type 5) contains quintuple non-synonymous SNPs (G689S, L845F, M908L, T958M and F1076L, marked in bold), novel non-synonymous SNP (Y541C), or synonymous SNPs (K44, L493, T529 and E1233) from 2016 to 2017. The frequency of each type was listed

### Phylogenetic analysis of the *pvmdr-1* gene

**Fig. 3:**
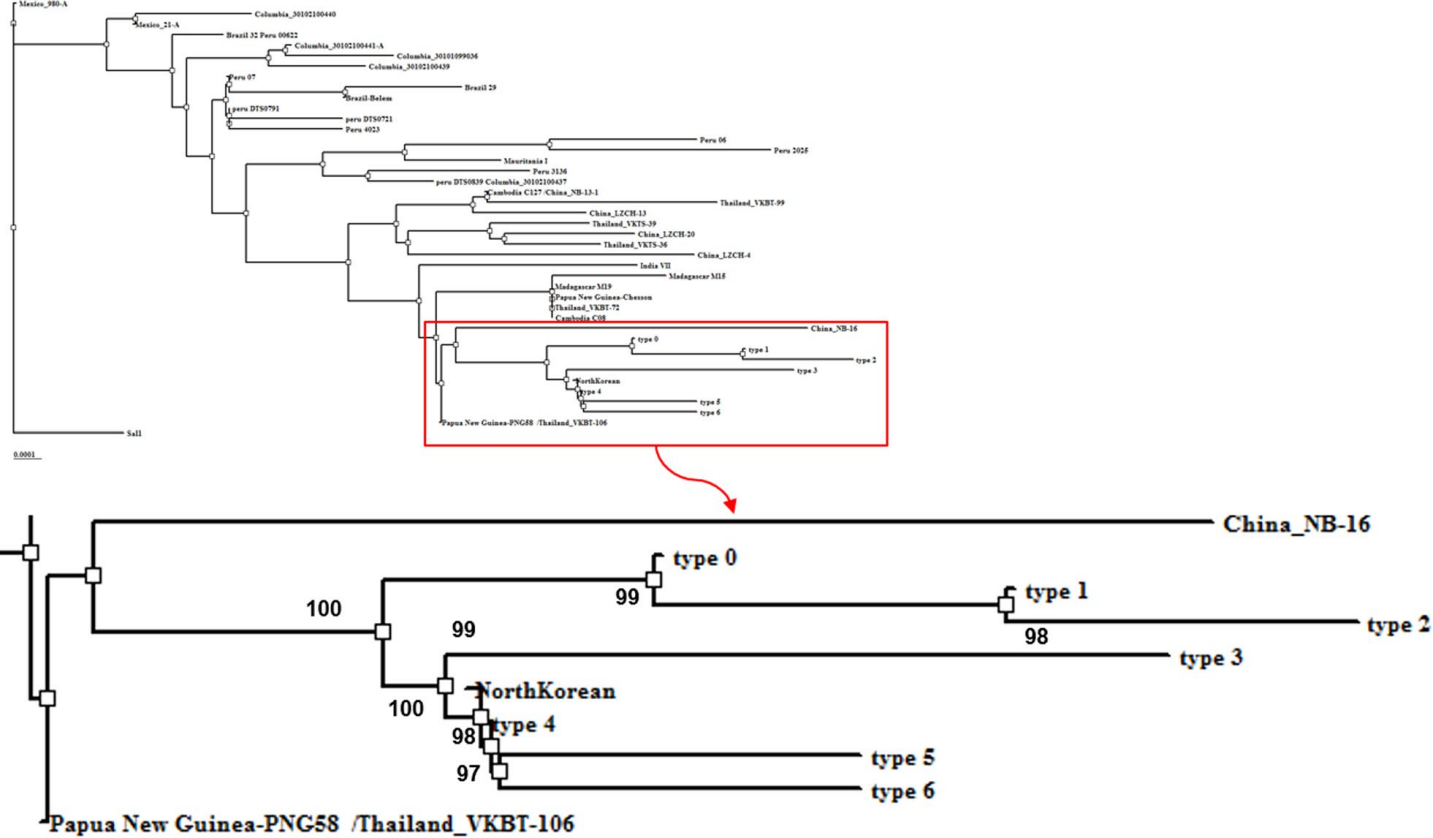
Phylogenetic analysis of 6 classified haplotypes via *pvmdr-1* SNP analysis of 73 *P. vivax* clinical samples. The sequences of *pvmdr-1* were aligned with that of Sal I strain (GenBank Accession# AY571984) and PlasmoDB by using BioEdit Sequence Alignment Editor. The aligned sequences were performed phylogenetic analysis by using 1000 bootstrap replications and the neighbour-joining method

The phylogenetic relationships on the overall *pvmdr-1* sequence were analysed and classified them into 6 haplotypes (Table 3). Type 4 sequence was identical to the *MDR* sequence in North Korean (*length* = 0.00001). The neighbourhood types of 6 haplotypes were identified as China_NB-16 and Papua New Guinea-PNG58/Thailand_VKBT-106 (Fig. 3).

## Discussion

*Plasmodium vivax*-endemic area is localized in the Gyeonggi province of the ROK near the DMZ. In this study, *pvmdr-1* SNPs of *P. vivax* were analysed using malaria-infected blood specimens in malaria-endemic regions including Goseong, Cheorwon, Yeonchen, Paju, Gimpo, and Hwacheon (Fig. 2). Since 1997, the ROK Army has conducted continuous malaria chemoprophylaxis (CQ and PQ) to prevent and reduce transmission of malaria for approximately 100,000 military personnel. Due to the massive chemoprophylaxis efforts, there is a consistent concern regarding CQR. According to various reports [5, 28], long-term or massive chemoprophylaxis could cause CQR. Thus, the surveillance system should be needed to estimate the risk of chemoprophylaxis-mediated drug resistance. ROK army also has been interested in analysing SNPs of drug resistant-related genes along with the implementation of chemoprophylaxis. Thus, the SNPs analysis of *pvmdr-1* against malaria-infected soldiers near chemoprophylaxis-executed malaria-endemic regions was investigated in clinical samples.

CQR has occasionally been observed in malaria-endemic regions that follow extensive chemoprophylaxis. Through preventive CQR study, CQR was confirmed in 2 of 484 enrolled patients [29]. CQR was also studied via the treatment responses of *P. vivax* malaria patients in the ROK monitored during 2003–2007 [28]. Until recently, it was reported that *P. vivax* resistance to CQ had emerged in South America and French Guiana [30, 31]. In the ROK, to date, most malaria patients in the military have been cured with malaria chemotherapy. However, issues with chemoprophylaxis-mediated resistance have recently been reported [6, 28]. For example, Yeom and colleagues suggested that malaria resistance to prophylactic agents could decrease CQ susceptibility, and they cited that the mass chemoprophylaxis with CQ in the ROK Army could contribute to this issue [5]. Therefore, the relationship between CQ susceptibility and chemoprophylaxis should also be investigated. To date, although CQR cases of *P. vivax* malaria have been reported in many other areas of the world, the drug resistance in malaria-endemic regions of ROK has been reported rarely [28]. However, the issue of drug insusceptibility to CQ and rapid transition of *pvmdr-1* SNPs has been raised [32].

In previous, Chung et al. identified four SNPs (F1076L, T529, E1233, and S1358) of *pvmdr-1* in malaria-endemic regions of ROK from 2011 to 2012 [11]. In this study, the Y541C, K44, L493, and T529 mutations centred around quintuple mutations (G698S, L845F, M908L, T958M, and F1076L) in the *pvmdr-1* gene were detected in malaria-infected military personnel of the ROK. Thus, it could be inferred that the rapid SNP transition of *pvmdr-1* in chemoprophylaxis-executed malaria-endemic regions has been occurring.

In the reports investigating the relationship between CQR and *pvmdr-1* SNP, Suwanarusk et al. revealed that the Y976F mutation was linked to CQR based on an increase in the IC_50_ value [33]. In another report, the F1076L mutation was linked to the Y976F SNP as a background mutation [34]. This study neither identified any SNPs at the Y976F position nor concluded whether Y976F was linked with F1076L. And it also showed no CQR with the Y541C mutation. Thus, it could be inferred that the Y541C mutation was not related to CQR.

Phylogenetic analysis showed that Type 4 (T529, G698S, L845F, M908L, T958M, and F1076L) was significantly related to the Type identified for North Koreans. In addition, a review by Chai indicated that a mosquito with blood from a malaria patient in North Korea near the DMZ flew south and infected Korean soldiers [6]. Based on this result, it is deduced that *P. vivax* malaria was introduced from North Korea, rather than as an influx from foreign countries.

Since 2016, to evaluate the risk from malaria chemoprophylaxis in the ROK, G6PD deficiency prevalence has been investigated [35], and to infer the risk of resistance potentially introduced by chemoprophylaxis, SNPs of *P. vivax* genes have been examined by the Armed forces Medical Command (AFMC) and the Armed forces Medical Research Institute (AFMRI) of ROK. This study focused SNPs of *pvmdr-1* against malaria-infected soldiers near chemoprophylaxis-executed malaria-endemic regions. However, for a more practical resistance analysis and effectiveness of malaria chemoprophylaxis, in vivo or ex vivo resistance studies via analysing malaria infection ratio or CQ metabolite under malaria chemoprophylaxis are needed via further research. Therefore, it is currently planned to perform these studies in cooperation with AFMC and KDCA (Korea Disease Control Agency).

## Conclusions

In this study, there were clinical samples that possessed various SNPs of *pvmdr-1*. Thus, it inferred that quintuple mutation (G698S, L845F, M908L, T958M, and F1076L) was a prevalent background mutation in malaria-infected military personnel of the ROK. Phylogenetic analysis indicated that the malaria type was close to the type seen in North Korean, indicating that *P. vivax* malaria in the ROK could have come from North Korea. Furthermore, consistent monitoring of chemoprophylaxis-linked *pvmdr-1* polymorphisms should be conducted to detect rapidly changed SNP profiling against *P. vivax* malaria in the ROK.

## Supplementary Information

**Additional file 1: Fig. S1.** Alignment and mapping data of *pvmdr-1* wild-type and mutant-type sequences in ROK army in 2016-2017. After the amplification of *pvmdr-1*using 73 *P. vivax* clinical samples, sequencing of PCR products was performed by using Big Dye™ Terminator v3.1 Cycle Sequencing Kit and ABI 3730XL Genetic Analyzer. Sequence analysis was performed using BioEdit Sequence Alignment Editor. The red box indicates the changed nucleotide in the alignment of *pvmdr-1* SNPs for 20 and 53 specimens in 2016 and 2017. MDRF-WT is used as a reference sequence of *pvmdr-1* gene (Gene Accession# AY571984).

## Data Availability

The datasets during and/or analysed during the current study available from the corresponding author(s) on reasonable request.
