# Supplementary figures and images for: Single nucleotide polymorphism analysis of pvmdr-1 in Plasmodium vivax isolated from military personnel of Republic of Korea in 2016 and 2017
Source: Malar J. 2022 Jun 28;21:205. doi: 10.1186/s12936-022-04214-6 (PMC9238087; doi:10.1186/s12936-022-04214-6)

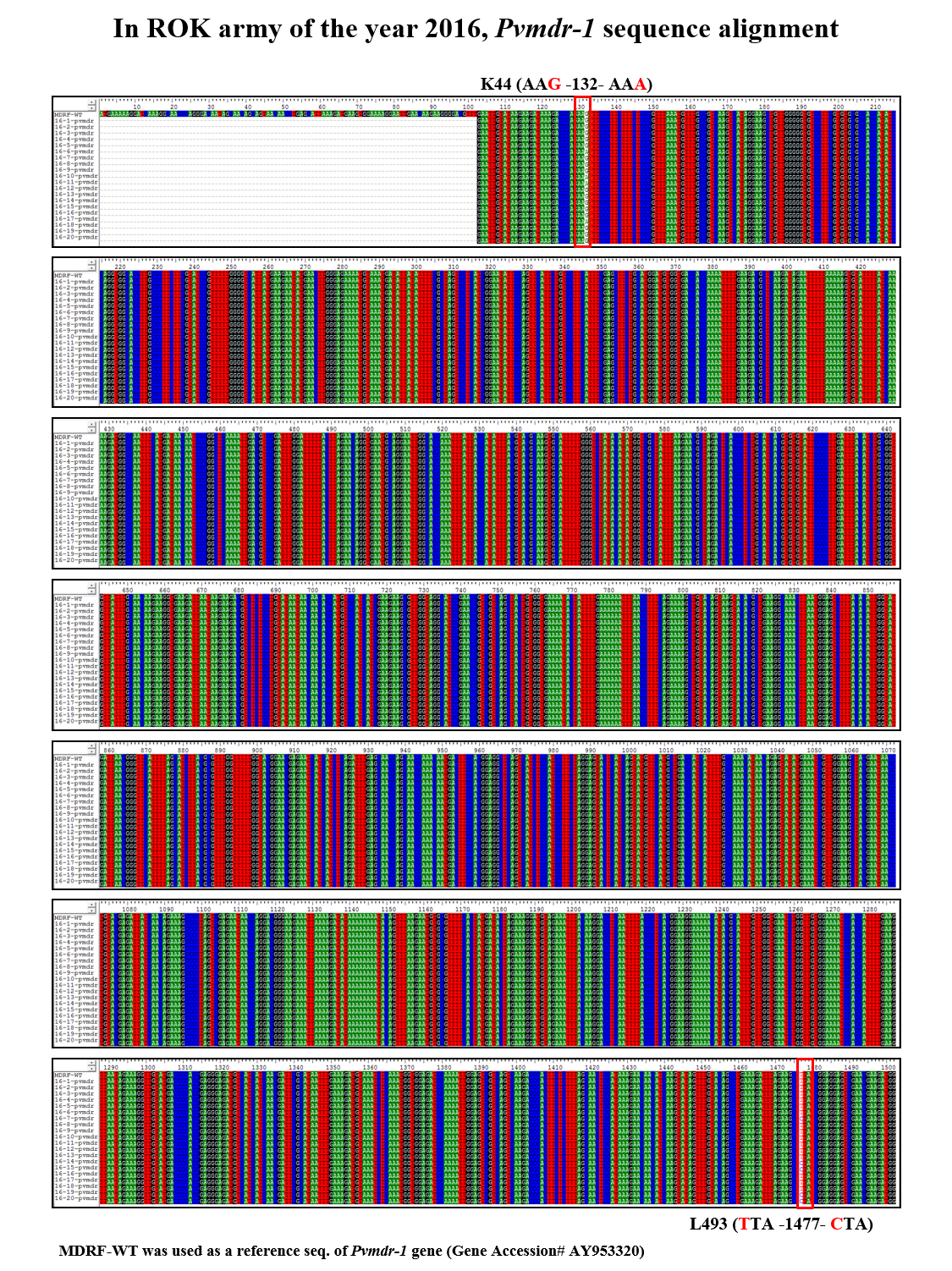

Supplement: Supplementary file 1 — Additional file 1: Fig. S1. Alignment and mapping data of pvmdr-1 wild-type and mutant-type sequences in ROK army in 2016-2017. After the amplification of pvmdr-1using 73 P. vivax clinical samples, sequencing of PCR products was performed by using Big Dye™ Terminator v3.1 Cycle Sequencing Kit and ABI 3730XL Genetic Analyzer. Sequence analysis was performed using BioEdit Sequence Alignment Editor. The red box indicates the changed nucleotide in the alignment of pvmdr-1 SNPs for 20 and 53 specimens in 2016 and 2017. MDRF-WT is used as a reference sequence of pvmdr-1 gene (Gene Accession# AY571984). [file 12936_2022_4214_MOESM1_ESM.zip › Fig.S1a_raw_images.tif]

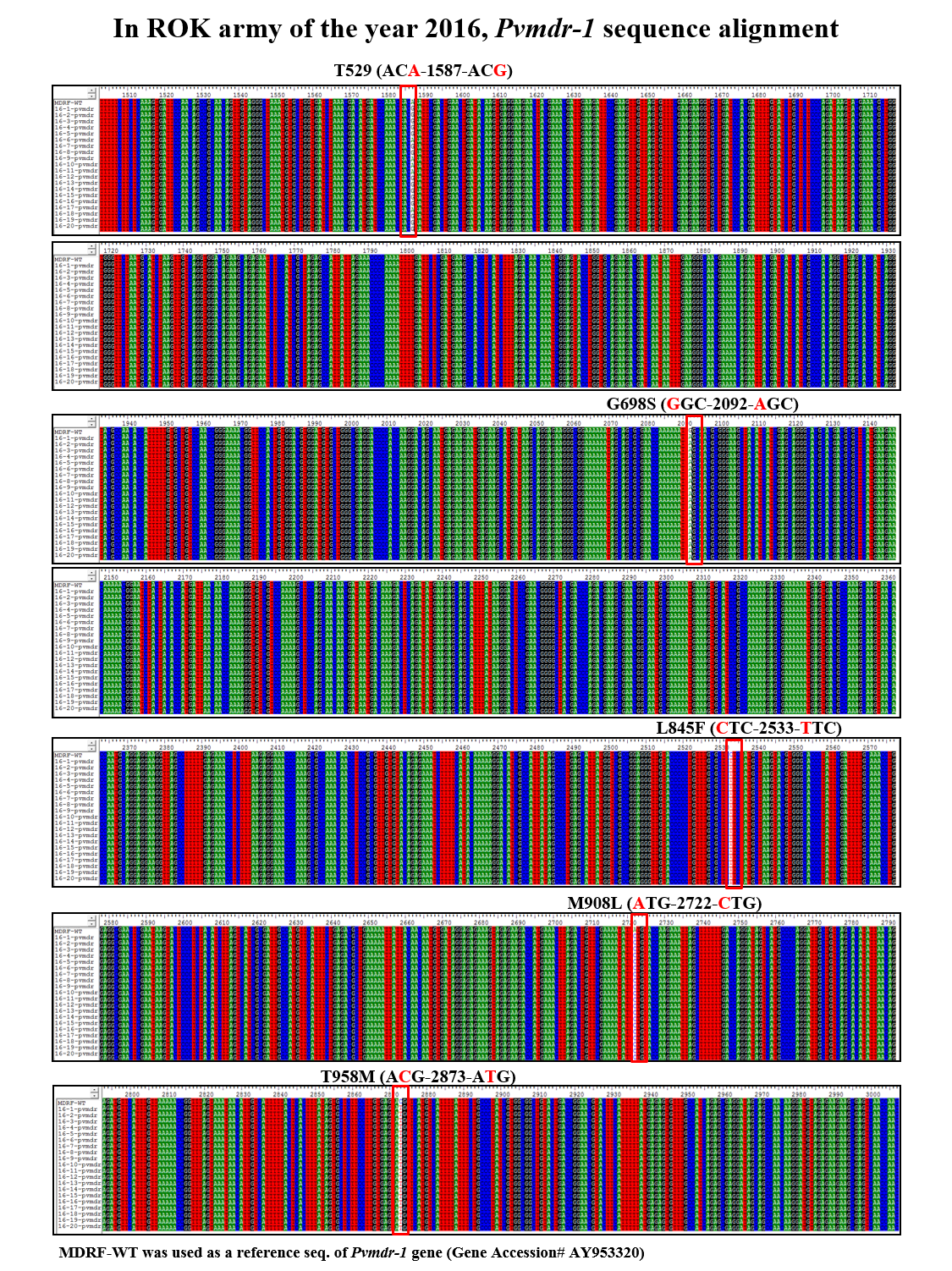

Supplement: Supplementary file 1 — Additional file 1: Fig. S1. Alignment and mapping data of pvmdr-1 wild-type and mutant-type sequences in ROK army in 2016-2017. After the amplification of pvmdr-1using 73 P. vivax clinical samples, sequencing of PCR products was performed by using Big Dye™ Terminator v3.1 Cycle Sequencing Kit and ABI 3730XL Genetic Analyzer. Sequence analysis was performed using BioEdit Sequence Alignment Editor. The red box indicates the changed nucleotide in the alignment of pvmdr-1 SNPs for 20 and 53 specimens in 2016 and 2017. MDRF-WT is used as a reference sequence of pvmdr-1 gene (Gene Accession# AY571984). [file 12936_2022_4214_MOESM1_ESM.zip › Fig.S1b_raw_images.tif]

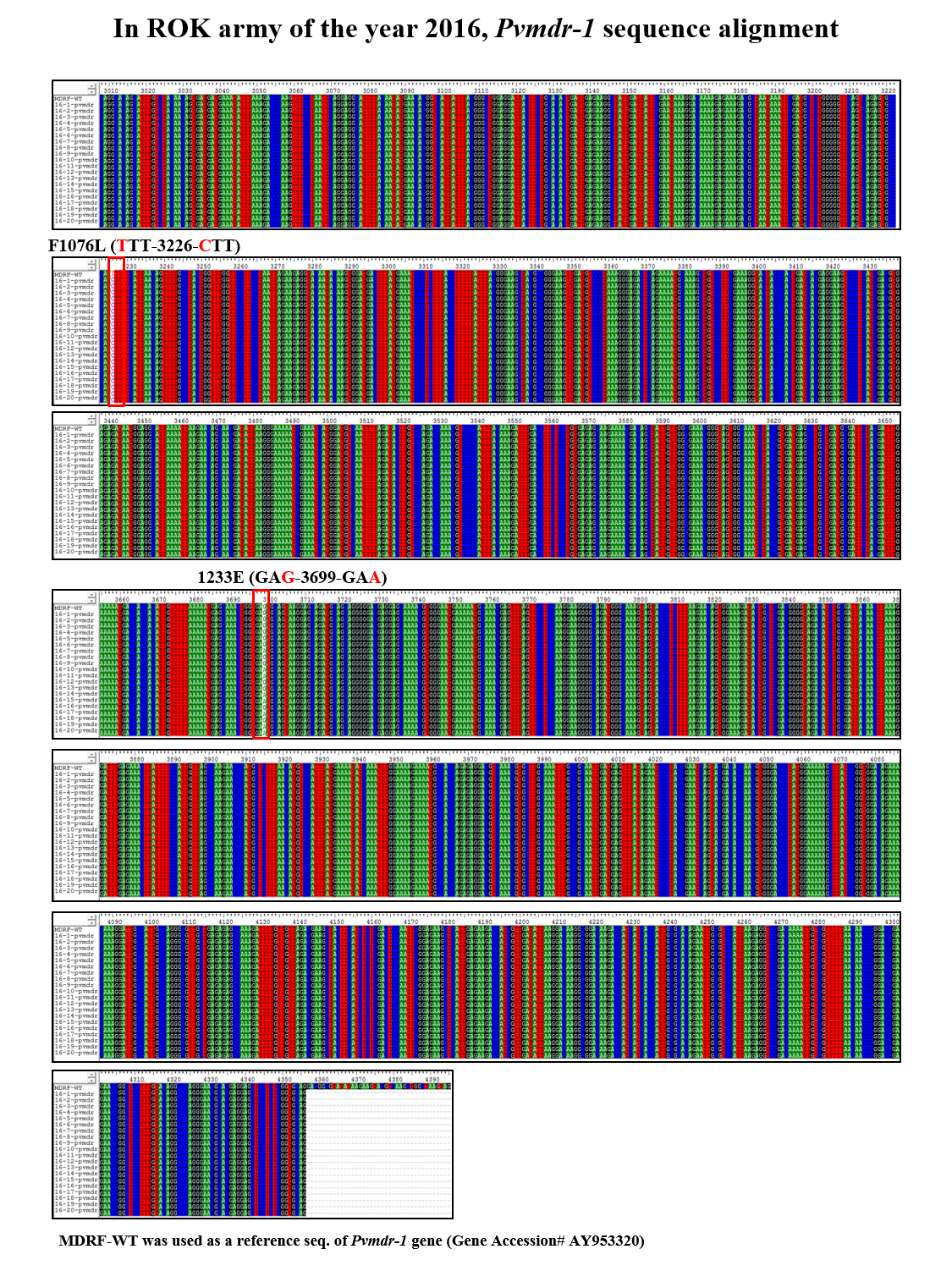

Supplement: Supplementary file 1 — Additional file 1: Fig. S1. Alignment and mapping data of pvmdr-1 wild-type and mutant-type sequences in ROK army in 2016-2017. After the amplification of pvmdr-1using 73 P. vivax clinical samples, sequencing of PCR products was performed by using Big Dye™ Terminator v3.1 Cycle Sequencing Kit and ABI 3730XL Genetic Analyzer. Sequence analysis was performed using BioEdit Sequence Alignment Editor. The red box indicates the changed nucleotide in the alignment of pvmdr-1 SNPs for 20 and 53 specimens in 2016 and 2017. MDRF-WT is used as a reference sequence of pvmdr-1 gene (Gene Accession# AY571984). [file 12936_2022_4214_MOESM1_ESM.zip › Fig.S1c_raw_images.tif]

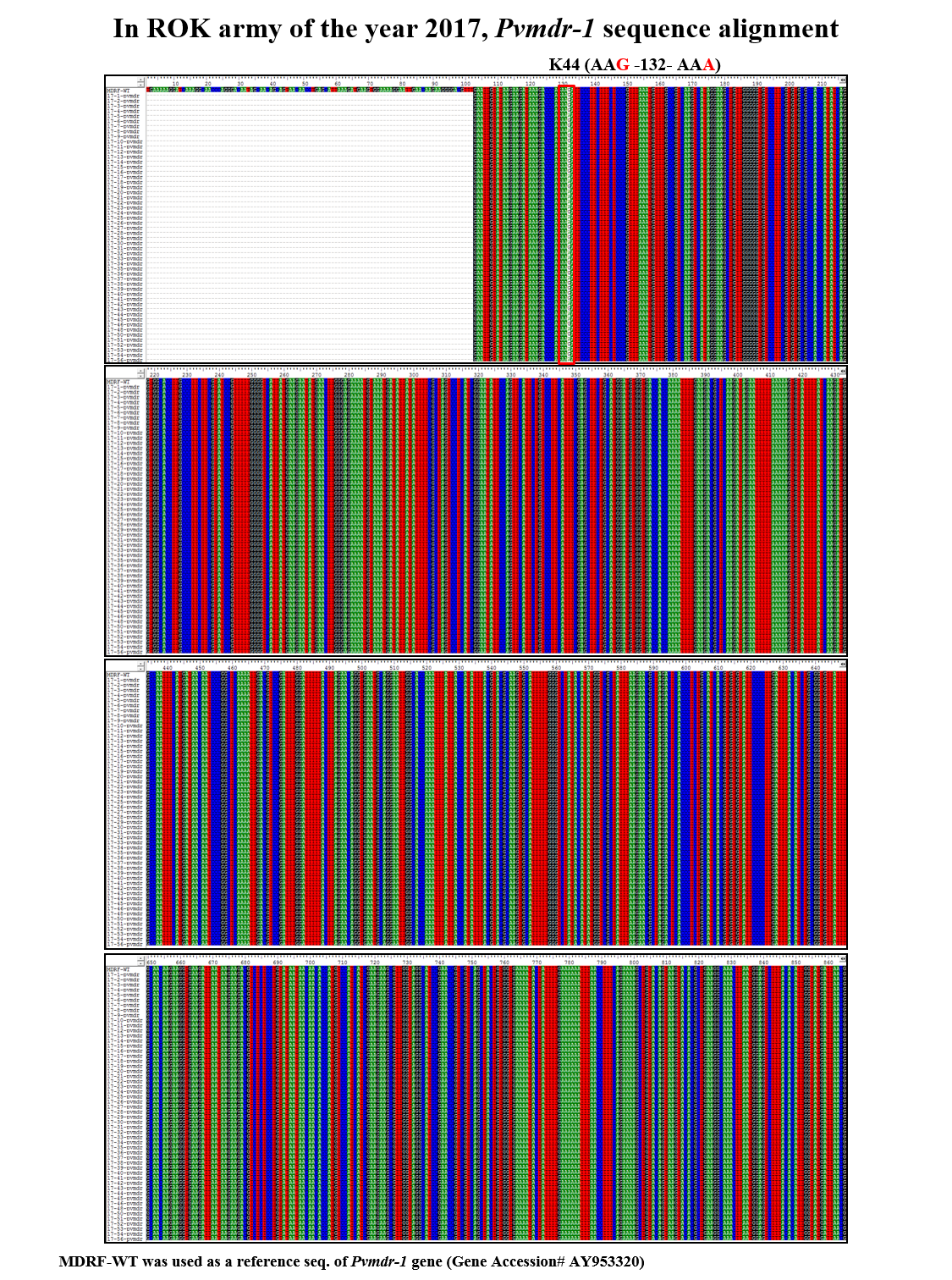

Supplement: Supplementary file 1 — Additional file 1: Fig. S1. Alignment and mapping data of pvmdr-1 wild-type and mutant-type sequences in ROK army in 2016-2017. After the amplification of pvmdr-1using 73 P. vivax clinical samples, sequencing of PCR products was performed by using Big Dye™ Terminator v3.1 Cycle Sequencing Kit and ABI 3730XL Genetic Analyzer. Sequence analysis was performed using BioEdit Sequence Alignment Editor. The red box indicates the changed nucleotide in the alignment of pvmdr-1 SNPs for 20 and 53 specimens in 2016 and 2017. MDRF-WT is used as a reference sequence of pvmdr-1 gene (Gene Accession# AY571984). [file 12936_2022_4214_MOESM1_ESM.zip › Fig.S1d_raw_images.tif]

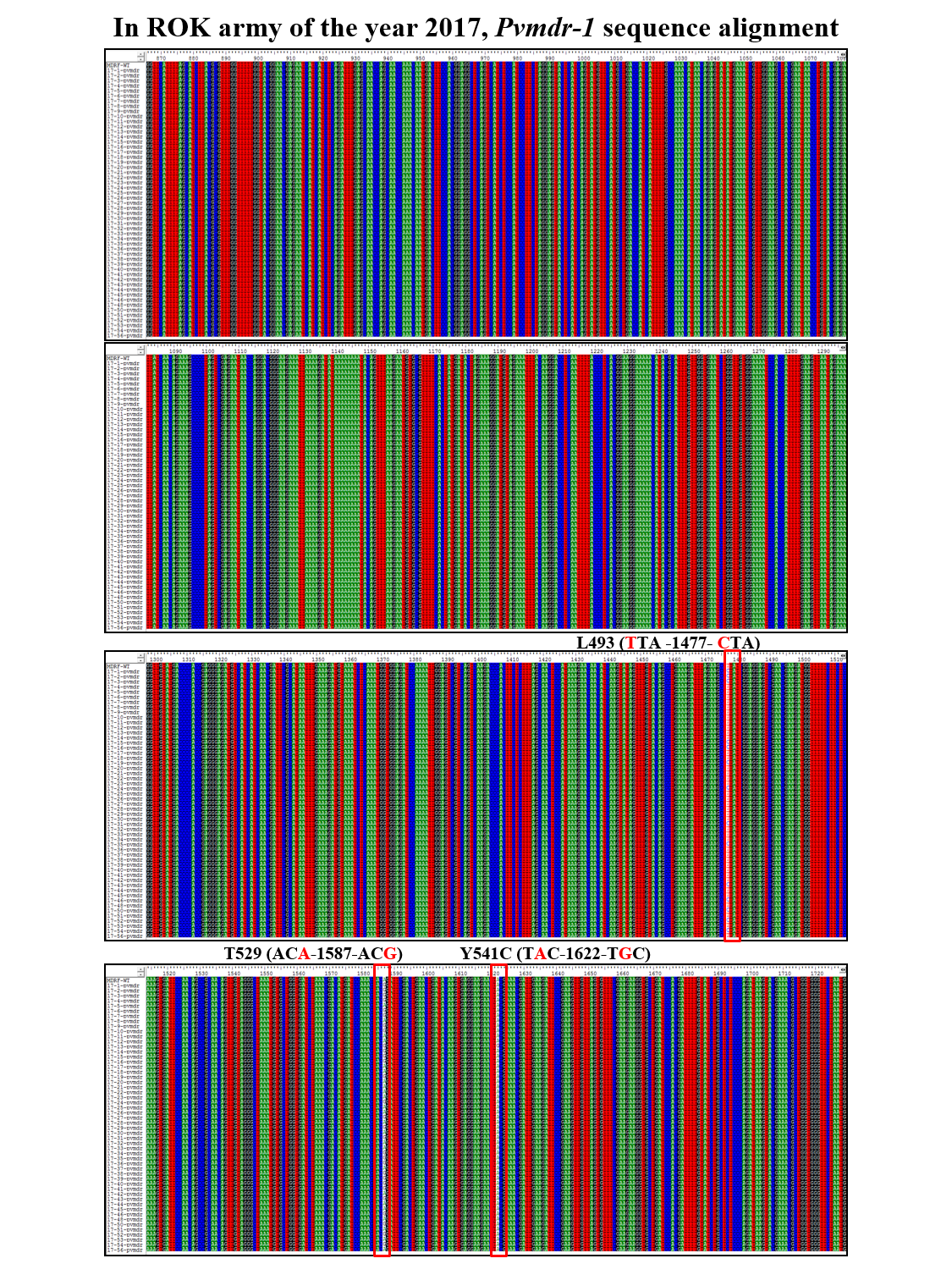

Supplement: Supplementary file 1 — Additional file 1: Fig. S1. Alignment and mapping data of pvmdr-1 wild-type and mutant-type sequences in ROK army in 2016-2017. After the amplification of pvmdr-1using 73 P. vivax clinical samples, sequencing of PCR products was performed by using Big Dye™ Terminator v3.1 Cycle Sequencing Kit and ABI 3730XL Genetic Analyzer. Sequence analysis was performed using BioEdit Sequence Alignment Editor. The red box indicates the changed nucleotide in the alignment of pvmdr-1 SNPs for 20 and 53 specimens in 2016 and 2017. MDRF-WT is used as a reference sequence of pvmdr-1 gene (Gene Accession# AY571984). [file 12936_2022_4214_MOESM1_ESM.zip › Fig.S1e_raw_images.tif]

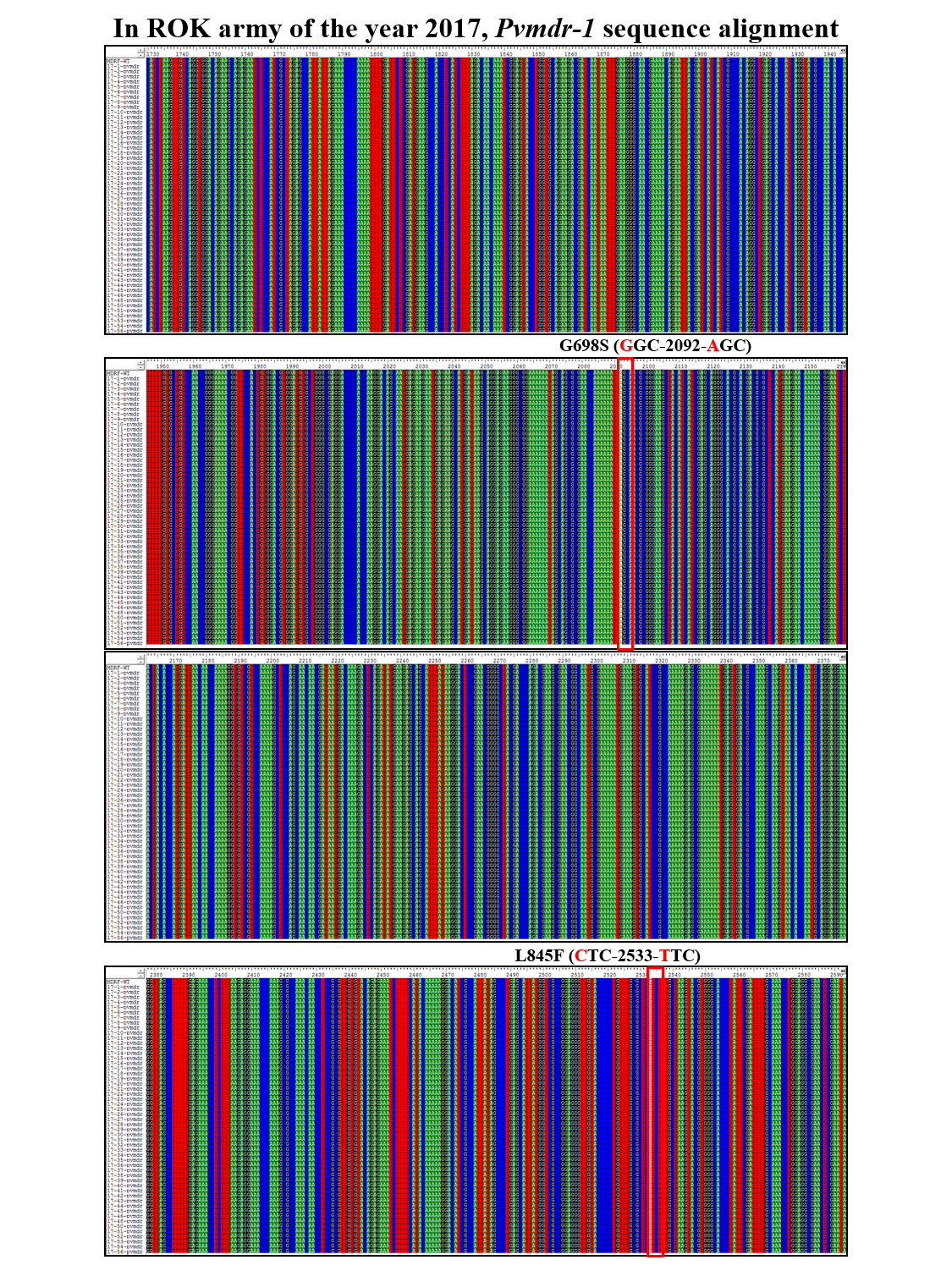

Supplement: Supplementary file 1 — Additional file 1: Fig. S1. Alignment and mapping data of pvmdr-1 wild-type and mutant-type sequences in ROK army in 2016-2017. After the amplification of pvmdr-1using 73 P. vivax clinical samples, sequencing of PCR products was performed by using Big Dye™ Terminator v3.1 Cycle Sequencing Kit and ABI 3730XL Genetic Analyzer. Sequence analysis was performed using BioEdit Sequence Alignment Editor. The red box indicates the changed nucleotide in the alignment of pvmdr-1 SNPs for 20 and 53 specimens in 2016 and 2017. MDRF-WT is used as a reference sequence of pvmdr-1 gene (Gene Accession# AY571984). [file 12936_2022_4214_MOESM1_ESM.zip › Fig.S1f_raw_images.tif]

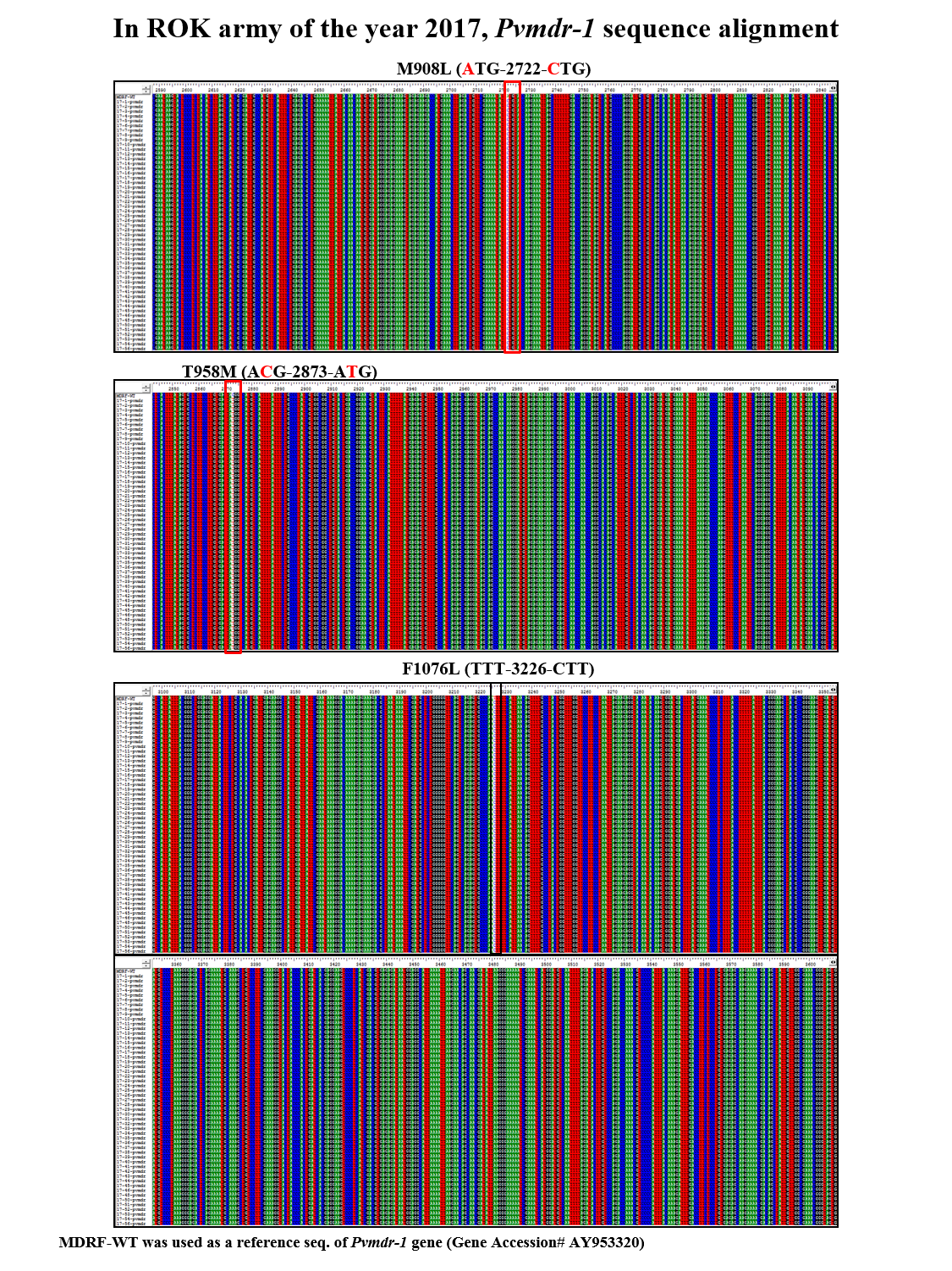

Supplement: Supplementary file 1 — Additional file 1: Fig. S1. Alignment and mapping data of pvmdr-1 wild-type and mutant-type sequences in ROK army in 2016-2017. After the amplification of pvmdr-1using 73 P. vivax clinical samples, sequencing of PCR products was performed by using Big Dye™ Terminator v3.1 Cycle Sequencing Kit and ABI 3730XL Genetic Analyzer. Sequence analysis was performed using BioEdit Sequence Alignment Editor. The red box indicates the changed nucleotide in the alignment of pvmdr-1 SNPs for 20 and 53 specimens in 2016 and 2017. MDRF-WT is used as a reference sequence of pvmdr-1 gene (Gene Accession# AY571984). [file 12936_2022_4214_MOESM1_ESM.zip › Fig.S1g_raw_images.tif]

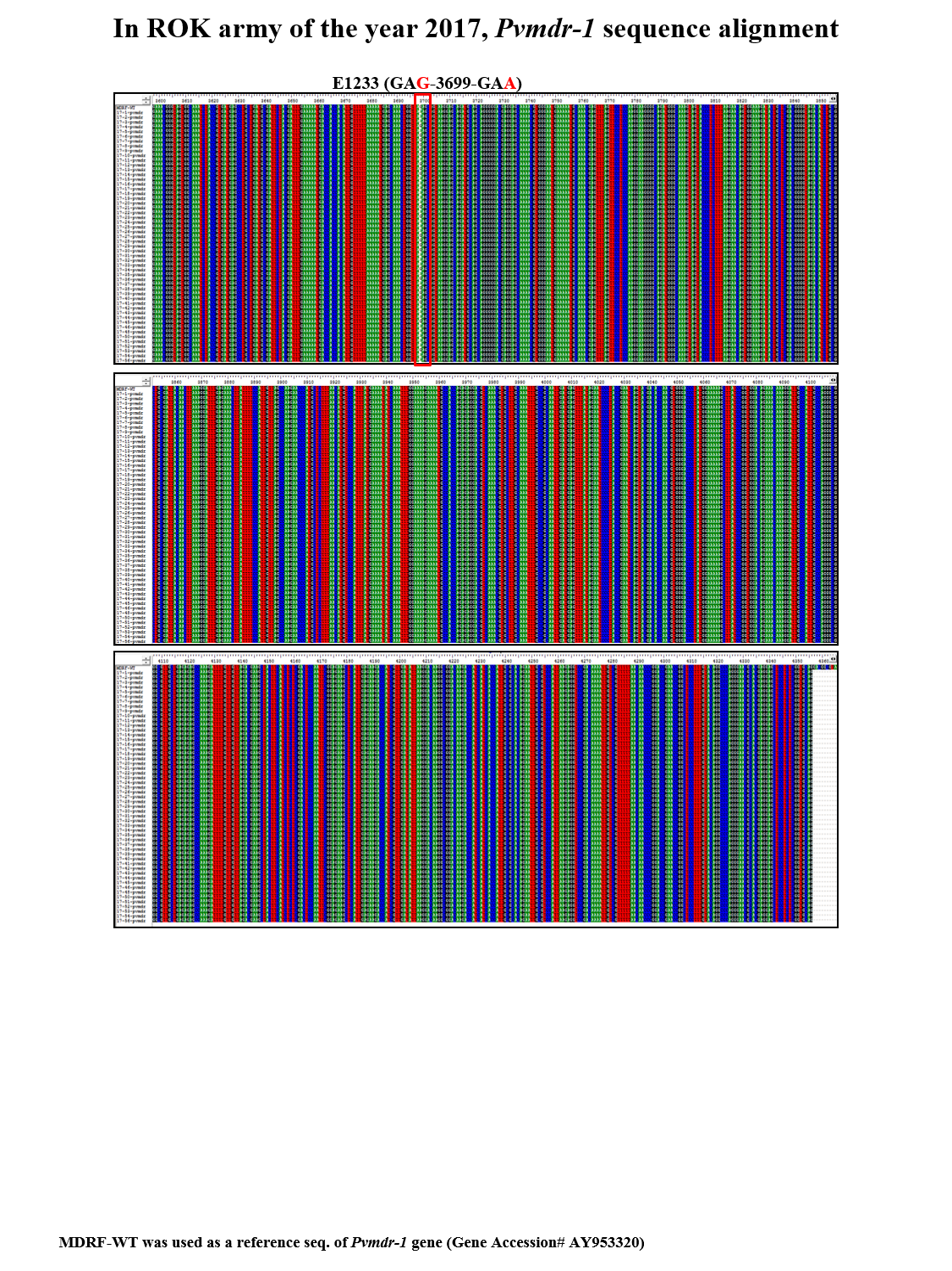

Supplement: Supplementary file 1 — Additional file 1: Fig. S1. Alignment and mapping data of pvmdr-1 wild-type and mutant-type sequences in ROK army in 2016-2017. After the amplification of pvmdr-1using 73 P. vivax clinical samples, sequencing of PCR products was performed by using Big Dye™ Terminator v3.1 Cycle Sequencing Kit and ABI 3730XL Genetic Analyzer. Sequence analysis was performed using BioEdit Sequence Alignment Editor. The red box indicates the changed nucleotide in the alignment of pvmdr-1 SNPs for 20 and 53 specimens in 2016 and 2017. MDRF-WT is used as a reference sequence of pvmdr-1 gene (Gene Accession# AY571984). [file 12936_2022_4214_MOESM1_ESM.zip › Fig.S1h_raw_images.tif]
